# Effect of Adjunctive Ozone Application Protocols on Dentin-Derived Growth Factor Release: An In Vitro Study

**DOI:** 10.3390/jcm15114277

**Published:** 2026-06-01

**Authors:** Sude Göbüt, Melis Oya Ateş, Ali Keleş, Fatma Avcıoğlu

**Affiliations:** 1 Department of Endodontics, Faculty of Dentistry, Bolu Abant Izzet Baysal University, Bolu 14030, Türkiye; sude.okur@ibu.edu.tr (S.G.); ali.keles@ibu.edu.tr (A.K.); 2 Muş Oral and Dental Health Center, Muş 49001, Türkiye; 3 Department of Medical Microbiology, Faculty of Medicine, Bolu Abant Izzet Baysal University, Bolu 14030, Türkiye; fatmaavcioglu@ibu.edu.tr

**Keywords:** regenerative endodontics, ozone, EDTA, growth factor release, micro-CT

## Abstract

**Background/Objectives:** Regenerative endodontic treatment (RET) depends on the release of dentin-derived bioactive molecules, which is commonly promoted by ethylenediaminetetraacetic acid (EDTA)-based dentin conditioning. However, whether adjunctive ozone delivery protocols can modify the measurable release of dentin-derived transforming growth factor beta 1 (TGF-β1) and insulin-like growth factor 1 (IGF-1) remains unclear. This study evaluated the effects of two adjunctive ozone application protocols used with chelation on dentin-derived TGF-β1 and IGF-1 release, without directly assessing the in situ activation or functional bioactivity of TGF-β1. **Methods:** Sixty-four freshly extracted human mandibular premolars were randomly assigned to four groups (*n* = 16). The experimental protocols were as follows: 17% EDTA alone (Group A), 17% EDTA followed by ozonated distilled water and ozone gas (Group B), ozonated 17% EDTA followed by ozone gas (Group C), and a negative control group. Root segments were standardized. In the experimental groups, all external surfaces were coated with nail varnish, leaving only the intracanal dentin surface exposed. In the negative control group, all surfaces were sealed. After ultrasonic activation, the specimens were incubated in phosphate-buffered saline (PBS) at 37 °C. PBS samples were collected on day 1 to evaluate early measurable growth factor release and on day 7 to assess short-term changes in detectable growth factor levels. TGF-β1 and IGF-1 levels were measured by ELISA and normalized to internal dentin surface area derived from micro-computed tomography (micro-CT) analysis. **Results:** No detectable growth factor values were observed in the negative control group. For TGF-β1, no significant intergroup difference was observed on day 1, whereas a significant difference was found on day 7 (*p* = 0.022). On day 7, the ozonated EDTA followed by ozone gas group showed approximately threefold higher surface-area-normalized TGF-β1 values than the EDTA followed by ozonated distilled water and ozone gas group (*p* = 0.018). TGF-β1 values increased from day 1 to day 7 in Groups A and C, whereas no significant temporal change was observed in Group B. IGF-1 values showed no significant intergroup or intragroup differences. **Conclusions:** Adjunctive ozone application showed a protocol-dependent effect on dentin-derived growth factor values, mainly for TGF-β1, while IGF-1 remained unaffected. The highest TGF-β1 values were observed when ozonated EDTA was followed by ozone gas. However, these in vitro findings indicate measurable growth factor release and should not be interpreted as direct evidence of TGF-β1 activation or clinical regenerative efficacy.

## 1. Introduction

In immature permanent teeth, caries, trauma, and developmental anomalies may lead to pulp necrosis and interruption of root development [1,2]. In teeth with open apices, conventional endodontic procedures are challenging due to thin root walls and a wide apical foramen, increasing the risk of material extrusion and root fracture [3,4,5]. Therefore, biologically based treatment approaches that promote continued root development and reinforcement of the root structure are preferred [6].

Regenerative endodontic treatment (RET) aims to restore or re-establish a functional dentin–pulp complex in immature necrotic teeth [7]. Its biological rationale is based on the interaction of the three key components of tissue engineering: stem cells, scaffolds, and signaling molecules [8]. The dentin matrix serves as a natural reservoir for growth factors, which can be released following demineralization induced by chelating agents or acids [9,10]. Thus, irrigation protocols used during RET are not only antimicrobial procedures but may also influence the release and availability of dentin-derived bioactive molecules [11,12].

During RET, low-concentration sodium hypochlorite (NaOCl) is primarily used for canal disinfection, whereas ethylenediaminetetraacetic acid (EDTA) is used as a final irrigant to condition dentin and improve its biological responsiveness [13]. Irrigation and chelation protocols not only provide antimicrobial effects but also regulate the release of dentin-derived biomolecules [14]. Although sodium hypochlorite is widely used for disinfection, it may adversely affect growth factor integrity [15]. In contrast, EDTA removes the smear layer, opens dentinal tubules, and enhances growth factor release [14,16]. Chelating agents facilitate this process by partially demineralizing superficial dentin and exposing matrix-bound bioactive proteins [17]. However, despite these advantages, the biological effects of EDTA may vary depending on the irrigation protocol and exposure conditions, and its demineralizing action may not result in uniform release of all dentin-derived growth factors [18]. In addition, recent evidence suggests that intracanal medicaments used in RET, such as calcium hydroxide and antibiotic-based pastes, may also influence dentin-derived growth factor release and should therefore be considered within the same biological framework [18,19]. For this reason, optimization of biologically active irrigation protocols remains an important area of investigation [20]. Although previous studies have demonstrated that EDTA and intracanal medicaments can affect dentin-derived growth factor release, it remains unclear whether adjunctive ozone application can further modify this response when combined with EDTA-based dentin conditioning. In this context, adjunctive ozone application has gained attention because of its antimicrobial potential and possible interaction with dentin conditioning procedures. Clarifying this issue is important because ozone is not intended to replace EDTA, but may serve as an adjunctive strategy within biologically oriented RET irrigation protocols.

Clinical studies on RET have reported high survival and success rates, together with radiographic evidence of continued root development, increased root wall thickness, and apical closure [21]. Accordingly, both the European Society of Endodontology [22] and the American Association of Endodontists [13] recommend the use of 17% EDTA following irrigation with low-concentration NaOCl as part of current RET protocols.

Various growth factors, including transforming growth factor beta 1 (TGF-β1) and insulin-like growth factor 1 (IGF-1), are released from demineralized dentin and regulate processes such as cell proliferation, differentiation, angiogenesis, and mineralization [20]. TGF-β1 plays a central role in odontoblastic differentiation and dentin formation, while IGF-1 contributes to mineralization and pulp cell survival [14,23,24,25]. Because of their established biological relevance in dentin–pulp regeneration, these two growth factors were selected as the outcome markers in the present study.

Ozone possesses antimicrobial and reparative properties and has been shown to modulate growth factor expression in dental and other biological tissues [26,27,28]. However, these findings should not be directly extrapolated to dentin-derived growth factor release, because direct evidence demonstrating ozone-mediated release or activation of matrix-bound TGF-β1 from human dentin remains limited. Therefore, the proposed ROS-related mechanism was considered only as a theoretical rationale for adjunctive ozone application, rather than as an established tissue-independent mechanism. Because dentin-derived growth factors are embedded within the mineralized extracellular matrix, their measurable release is expected to depend primarily on dentin conditioning, matrix exposure, and the physicochemical interaction between adjunctive agents and EDTA-conditioned dentin surfaces [11]. EDTA was therefore considered the main agent responsible for dentin demineralization and exposure of matrix-bound bioactive molecules. The mechanistic hypothesis for adjunctive ozone was that ozone-related oxidative effects might further modify the EDTA-conditioned dentin surface or influence the availability of matrix-associated proteins, thereby potentially altering the measurable release of TGF-β1 and IGF-1. However, whether different adjunctive ozone delivery protocols produce distinct effects on dentin-derived growth factor release after EDTA-based conditioning remains unclear. Based on the available literature, there is limited quantitative evidence comparing adjunctive ozone delivery protocols in terms of their effect on dentin-derived TGF-β1 and IGF-1 release. In this context, the present study aimed to compare two adjunctive ozone application protocols used in combination with chelation and to determine whether differences in ozone delivery influence the release of dentin-derived TGF-β1 and IGF-1. The null hypothesis was that adjunctive ozone application protocols would not affect dentin-derived TGF-β1 or IGF-1 release.

## 2. Materials and Methods

### 2.1. Ethics Approval

This laboratory study was conducted in accordance with the ethical principles of the Declaration of Helsinki and its subsequent amendments. The study was reported in accordance with the Preferred Reporting Items for Laboratory Studies in Endodontology 2021 Guidelines [29]. Ethical approval was obtained from Bolu Abant Izzet Baysal University Non-Interventional Clinical Research Ethics Committee (Decision No. 2024/186; approval date: 17 July 2024). Written informed consent was obtained from all patients before the use of the extracted teeth for research purposes. The extracted teeth were collected after ethics committee approval between August 2024 and September 2024. Tooth extraction was performed for clinical reasons unrelated to the present study.

### 2.2. Determination of Sample Size

Sample size estimation was performed using G*Power software (version 3.1.9.7; Heinrich Heine University, Düsseldorf, Germany), based on a previous study with a similar experimental design [30]. For TGF-β1, an effect size of Cohen’s f = 0.600 was used. With α = 0.05 and power = 0.95, the analysis indicated that a minimum of 16 specimens was required for each experimental group. An additional calculation based on IGF-1 using Cohen’s f = 0.763 indicated that a minimum of 10 specimens was required for each experimental group. Since the TGF-β1-based calculation required the larger sample size, 16 teeth were included in each experimental group.

### 2.3. Inclusion Criteria of the Teeth

Freshly extracted human mandibular premolars with a single root and a single canal, collected from healthy patients aged 18–40 years, were included in this study. Teeth with incomplete root formation, caries, cracks, fractures, internal or external resorption, calcification, or anatomical variations were excluded. All specimens were initially inspected under a dental operating microscope at ×20 magnification (Zumax Medical Co., Ltd., Suzhou, China) to identify visible cracks and other structural defects. Standardized digital periapical radiographs were then obtained in both buccolingual and mesiodistal directions to confirm the presence of a single canal and the absence of additional canals or resorptive defects. Single-rooted mandibular premolars were selected to obtain a standardized experimental model with single-canal anatomy, to reduce anatomical variability among specimens, and to allow more consistent internal surface area normalization using micro-computed tomography (micro-CT).

### 2.4. Preparation and Standardization of Root Segments

Following extraction, soft tissue remnants and surface debris were carefully removed from the root surfaces with periodontal curettes. The teeth were rinsed with phosphate-buffered saline (PBS; Gündüz Kimya, Istanbul, Türkiye) and stored in 0.5% chloramine-T solution (BMV Kimya, Istanbul, Türkiye) until use. The storage medium was replaced with distilled water 24 h before experimentation. Autoclaving was avoided because heat may alter dentin structure and compromise the integrity of proteins relevant to growth factor analysis.

The teeth were numbered from 1 to 64 and randomly allocated to four groups (*n* = 16) using a computer-generated random sequence obtained from www.random.org (accessed on 25 September 2024). After decoronation, the roots were standardized to a canal length of 10 mm measured from the apex [31]. To simulate a wide apical foramen, canal preparation was performed using Gates-Glidden burs (#1–4, VDW, Munich, Germany) [32]. The final canal preparation protocol was therefore standardized across all groups. However, because extracted human teeth may show inherent anatomical variability, micro-CT analysis was performed after specimen preparation to quantify root canal volume and internal root canal surface area for each specimen. Internal root canal surface area was subsequently used for normalization of growth factor values.

The root segments were then embedded in Eppendorf tubes using a silicone-based impression material (Zetaplus L Intro Kit; Zhermack S.p.A., Rovigo, Italy). This procedure was performed to provide a standardized apical barrier and to limit apical extrusion of chelating agents during the experimental procedures, thereby approximating clinical irrigation conditions [30]. In the experimental groups, all external root surfaces, including the apical and coronal surfaces, were coated with nail varnish, leaving only the intracanal dentin surface exposed. In the negative control group, all surfaces, including the intracanal dentin surface, were sealed with nail varnish. This setup was designed to standardize the source of measurable dentin-derived growth factor release from the intracanal dentin surface rather than to reproduce the full biological and mechanical complexity of the periapical tissue environment. However, its potential influence on diffusion should be considered when interpreting the findings.

### 2.5. Experimental Irrigation and Ozone Application Protocols

All irrigation, ozone application, and ultrasonic activation procedures were performed by the same experienced operator (S.G.) throughout the study to minimize operator-related variability. Before the experimental procedures, the operator was calibrated to the standardized protocol, including irrigant volume, needle positioning, ozone application time, and ultrasonic activation movements. However, intra-operator reproducibility was not assessed using a separate quantitative reproducibility test.

In all experimental specimens, an initial irrigation protocol was performed to simulate the disinfection step of RET. Irrigation was performed with 20 mL of freshly prepared 1.5% NaOCl, obtained by diluting 3% NaOCl (CanalPro NaOCl 3%; Coltene/Whaledent AG, Altstätten, Switzerland) with sterile distilled water, followed by 20 mL of saline solution using a 30-gauge single-use endodontic irrigation needle (Medic, Galaxy Dental, Istanbul, Türkiye) positioned approximately 1 mm short of the root apex [13]. Subsequently, group-specific dentin conditioning protocols were applied to compare a standard chelation protocol with two adjunctive ozone delivery strategies used in combination with chelation.

Group A: Irrigation was performed with 20 mL of 17% EDTA solution (Saver, Prime Dental, Maharashtra, India).

Group B: After irrigation with 20 mL of 17% EDTA, irrigation was performed with 20 mL of ozonated distilled water (30 L/h flow rate, 80 μg/mL concentration, 5 min, Ozonette Ozone Generator, SEDECAL, Madrid, Spain), and then ozone gas (800 mbar) was applied into the canal for 60 s.

Group C: Irrigation was performed with 20 mL of ozonated EDTA (17%, 30 L/h flow, 80 μg/mL concentration, 5 min), and ozone gas (800 mbar) was applied into the canal for 60 s.

The ozone parameters were selected to provide a standardized and reproducible adjunctive application. Since no validated endodontic standard could be identified for the direct pre-ozonation of irrigants, the concentration of 80 μg/mL, 30 L/h flow rate, and 5 min ozonation period were used as standardized experimental settings within the operational range of the ozone generator. Ozone gas application at 800 mbar for 60 s was selected to represent a short, clinically feasible and reproducible intracanal adjunctive protocol. These parameters should therefore be interpreted as experimental settings rather than as an established clinical standard.

Negative control group: All surfaces, including the intracanal dentin surface, were coated with nail varnish, and no irrigation or activation procedure was performed.

Ultrasonic activation was applied in all experimental groups as a standardized delivery method to improve irrigant contact with intracanal dentin and to reflect an activated irrigation approach [30,31]. Because ultrasonic activation was applied uniformly across all experimental groups, it was not investigated as an independent variable, and its separate effect on dentin-derived growth factor release could not be determined in the present study. During irrigation, continuous ultrasonic activation was performed using an ultrasonic device (P5 Newtron XS, Satelec Acteon, Mérignac, France) and an ultrasonic activation tip (IF43727, K 20/25, Satelec, Acteon, Mérignac, France) with 4–5 mm up-and-down movements [30]. After the procedure, the canals were dried with sterile paper points (Meta Dental Co., Ltd., Chongiu City, Republic of Korea), and each specimen was placed into sterile Eppendorf tubes containing 1 mL of PBS and stored in an incubator at 37 °C.

### 2.6. Sample Collection and ELISA Analysis

On day 1 and day 7 after irrigation, a 400 μL aliquot of PBS was collected from each tube, transferred into sterile Eppendorf tubes, and stored at −80 °C. The PBS medium was not completely renewed after the day 1 collection. After removal of the day 1 aliquot, the specimens were returned to incubation in the remaining PBS until the day 7 collection. Therefore, the day 1 and day 7 measurements should be interpreted as detectable growth factor values at two sampling time points under the present incubation conditions, rather than as fully independent or cumulative release measurements. All Enzyme-Linked Immunosorbent Assay (ELISA) analyses were carried out in the Medical Microbiology Laboratory, Faculty of Medicine, Bolu Abant Izzet Baysal University. Commercial kits were used for TGF-β1 (Ref: E-EL-0162, sensitivity: 0.1 ng/mL, range: 0.16–10 ng/mL) and IGF-1 (Ref: E-EL-H0086, sensitivity: 0.94 ng/mL, range: 1.56–100 ng/mL) (Elabscience, Houston, TX, USA). Because TGF-β1 is present in a latent form in biological samples, an activation step was performed before analysis in accordance with the manufacturer’s protocol. Briefly, the collected PBS samples designated for TGF-β1 measurement were subjected to the activation procedure recommended by the kit manufacturer to convert latent TGF-β1 into its immunoreactive form. This procedure resulted in a 10-fold dilution of the samples. After activation and dilution, the TGF-β1 assay was performed according to the manufacturer’s instructions, following the same ELISA workflow used for IGF-1. All samples were assayed in duplicate, and the mean value of the duplicate wells was used for statistical analysis. Standard curves and kit controls were evaluated for each assay plate to monitor assay performance. The results were recorded using an ELISA reader at 450 nm. To minimize measurement bias, the ELISA assessment was performed in a blinded manner. Samples were coded by researchers involved in specimen preparation and experimental procedures, and the investigator responsible for the ELISA analysis was unaware of the group allocation during measurement.

### 2.7. Micro-CT Analysis

**Figure 1:**
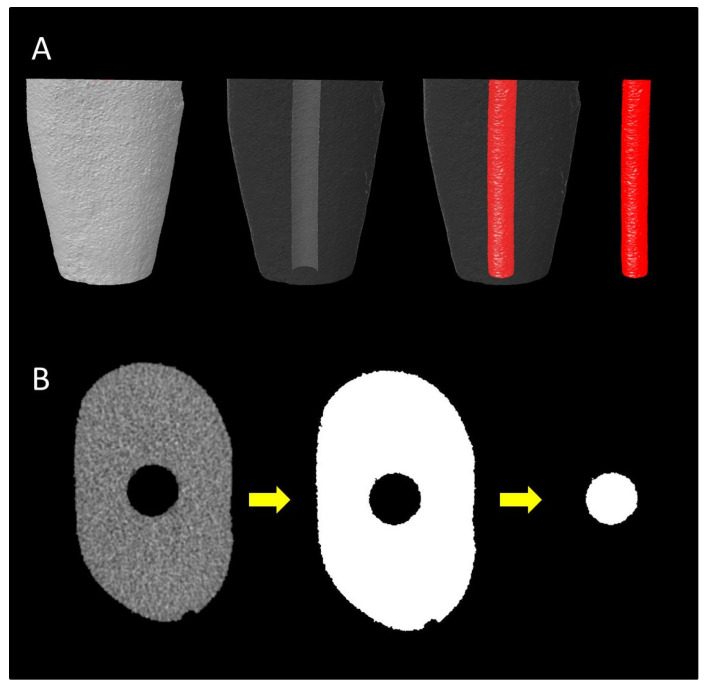
Representative micro-CT analysis workflow. (**A**) Three-dimensional segmentation of the root canal lumen, shown in red. (**B**) Histogram-based grayscale thresholding of cross-sectional images used to distinguish the canal lumen from the surrounding dentin and to define the region of interest for internal surface area calculation.

The specimens were scanned using a micro-CT device (SkyScan 1172; Bruker microCT, Kontich, Belgium). The scan parameters were set to 85 kV, 118 μA, 13.68 µm pixel size, 180° rotation, with a 0.6° rotation step and an aluminum–copper filter. The images were reconstructed using NRecon software v.1.7.4.6 (Bruker-microCT, Kontich, Belgium) with a 35% beam-hardening correction, a ring artifact correction of 5, and a smoothing of 2. The reconstructed images were analyzed using CTAn software v. 1.20.8.0 (Bruker-microCT, Kontich, Belgium). The region of interest was defined along the standardized 10 mm root segment used in the experiment. Root canal space segmentation was performed using histogram-based grayscale thresholding to distinguish the low-density root canal lumen from the surrounding dentin. The thresholding criteria were applied consistently to all specimens, and the accuracy of canal boundary detection was visually checked on representative cross-sectional slices before morphometric measurements. After segmentation, the canal lumen was isolated from the surrounding dentin, and root canal volume (mm^3^) and internal root canal surface area (mm^2^) were calculated using the morphometric tools of the software (Figure 1). The internal root canal surface area values were used to normalize the amount of dentin-derived growth factors released from each specimen.

### 2.8. Calculation of the Results

ELISA measurements were obtained as growth factor concentrations expressed in ng/mL. For each time point, the measured concentration was multiplied by the collected PBS aliquot volume of 400 μL, corresponding to 0.4 mL, to calculate the amount of growth factor present in the collected sample:

Growth factor amount (ng) = ELISA concentration (ng/mL) × 0.4 mL.

This amount was then divided by the micro-CT-derived internal root canal surface area (mm^2^) of each specimen to obtain the surface-area-normalized growth factor value:

Normalized growth factor value (ng/mm^2^) = growth factor amount (ng)/internal root canal surface area (mm^2^).

This normalization step was performed to minimize the influence of interspecimen variation in internal root canal surface area.

### 2.9. Statistical Analysis

Descriptive statistics were calculated for all variables and are presented as mean ± standard deviation and median with 25th and 75th percentiles, as appropriate. The normality of the data was evaluated using the Shapiro–Wilk test. Because the normality assumption was not met for several variables, nonparametric tests were used for inferential comparisons. Potential outliers were evaluated by graphical inspection of the distributions and by reviewing values outside the expected interquartile range. Observations were not excluded unless they could be attributed to a clear measurement or data-entry error. Mean ± standard deviation values were reported only as descriptive measures to show data dispersion and to facilitate comparison with previous studies, whereas the inferential statistical interpretation was based on nonparametric tests.

For intergroup comparisons of TGF-β1 and IGF-1 values at each time point, the Kruskal–Wallis test was used. The Kruskal–Wallis test was also used to compare root canal volume and internal root canal surface area among the experimental groups. When a significant intergroup difference was detected, Bonferroni-corrected post hoc pairwise comparisons were performed to identify the source of the difference.

For within-group temporal comparisons between day 1 and day 7, the Wilcoxon signed-rank test was used separately for each group and each growth factor. Spearman correlation analysis was used to assess relationships among non-normally distributed continuous growth factor measurements, including TGF-β1 and IGF-1 values at different time points. All analyses were performed using IBM SPSS Statistics version 27.0 (IBM Corp., Armonk, NY, USA). The significance level was set at *p* < 0.05.

## 3. Results

In the negative control group, in which all surfaces, including the intracanal dentin surface, were coated with nail varnish, no growth factor release was detected on day 1 or day 7. Therefore, the detectable TGF-β1 and IGF-1 values in the experimental groups were interpreted as being primarily related to release from the exposed intracanal dentin surface (Table 1).

**Figure 2:**
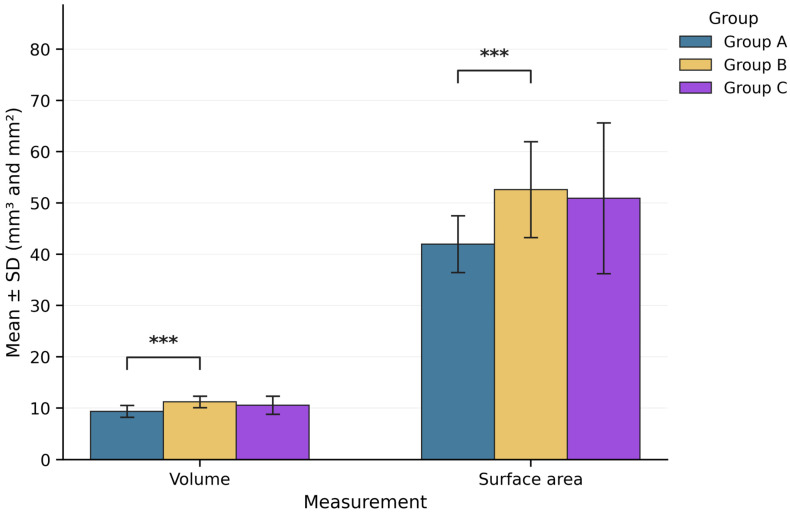
Comparison of micro-CT-derived root canal volume (mm^3^) and internal dentin surface area (mm^2^) among the study groups. Bars represent mean values, and error bars indicate standard deviation. Asterisks indicate statistically significant differences between groups.

Micro-CT analysis showed significant intergroup differences in root canal volume and internal dentin surface area (both *p* < 0.001). Post hoc comparisons demonstrated that Group B had significantly greater canal volume than Group A (*p* < 0.001) and significantly greater dentin surface area than Group A (*p* = 0.001), whereas no other pairwise differences were observed (*p* > 0.05). Because growth factor release was evaluated in relation to the exposed intracanal dentin surface, ELISA-derived values were normalized to the micro-CT-derived internal dentin surface area and are presented as ng/mm^2^ (Table 2, Figure 2). However, these baseline differences in canal volume and surface area should be considered when interpreting the growth factor results.

**Figure 3:**
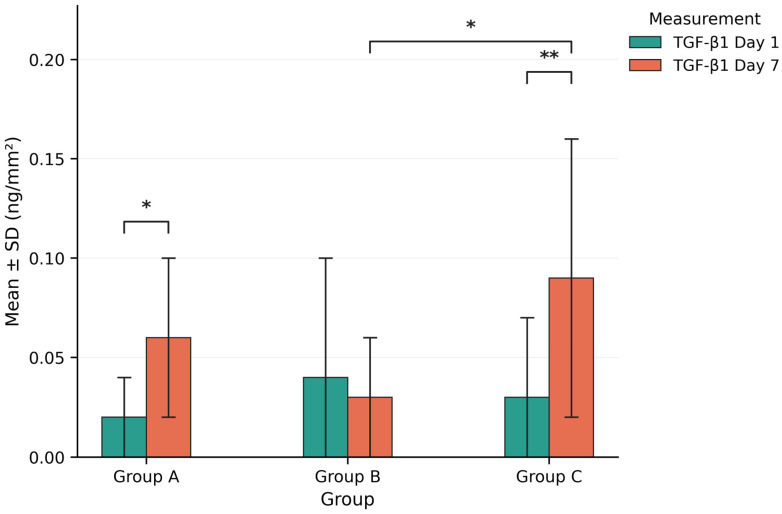
Surface-area-normalized TGF-β1 release values on day 1 and day 7 among the study groups. Bars represent mean values, and error bars indicate standard deviation. * indicates statistically significant differences for the bracketed comparisons in Group A between day 1 and day 7 and between Group B and Group C on day 7. ** indicates a statistically significant within-group difference in Group C between day 1 and day 7.

TGF-β1 release values are presented in Table 1 and Figure 3. Overall, TGF-β1 showed a significant time-dependent increase in Groups A and C, whereas no significant temporal change was observed in Group B. No significant intergroup difference was detected on day 1, but a significant intergroup difference was observed on day 7. This difference was mainly attributable to higher TGF-β1 values in Group C than in Group B after Bonferroni correction. No significant differences were observed between Group A and the other experimental groups. These findings indicate that the increase in TGF-β1 was protocol-dependent and was observed particularly when ozonated EDTA was followed by ozone gas application.

**Figure 4:**
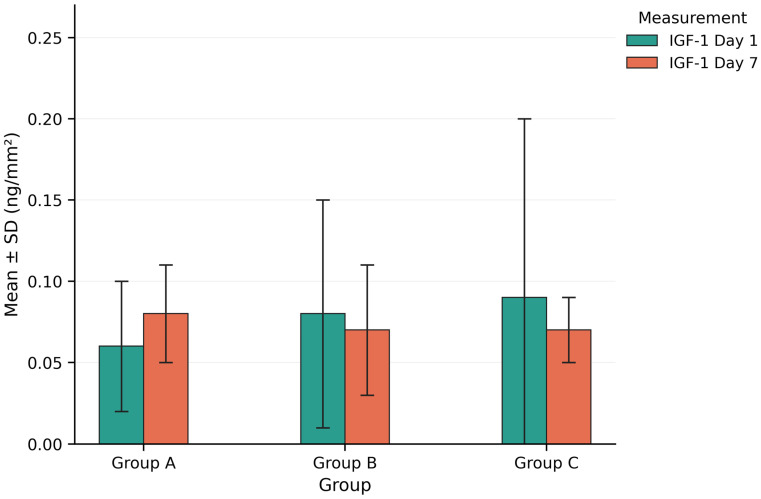
Surface-area-normalized IGF-1 values on day 1 and day 7 according to study group. Bars represent mean values, and error bars indicate standard deviation.

IGF-1 release values are presented in Table 1 and Figure 4. Overall, IGF-1 values remained low across all experimental groups and time points. No significant intergroup differences were observed on either day 1 or day 7, and no significant within-group temporal changes were detected. Because the measured IGF-1 values were consistently low, a possible floor effect should be considered when interpreting the absence of significant differences. Therefore, the present findings suggest that the tested protocols did not produce a detectable change in IGF-1 release under the present experimental conditions.

Spearman correlation analysis performed on the pooled dataset showed significant positive correlation between IGF-1 day 1 and TGF-β1 day 1, and between TGF-β1 day 1 and TGF-β1 day 7 (Table 3). However, because these analyses were performed on the pooled dataset, the observed associations may partly reflect group-level structure rather than direct biological relationships between biomarkers. Therefore, these correlations should be interpreted as exploratory findings. No other pooled correlations were statistically significant.

Group-specific Spearman correlation analyses are presented in Table 4. Several statistically significant positive correlations were observed within individual groups, including associations between IGF-1 day 1 and TGF-β1 day 1 in Groups A and C, between TGF-β1 day 1 and TGF-β1 day 7 in Groups B and C, and between IGF-1 day 7 and TGF-β1 day 7 in Group C. However, because multiple correlations were tested within each group, these findings should be interpreted cautiously as exploratory and hypothesis-generating rather than confirmatory evidence of direct biological relationships. Therefore, no definitive mechanistic interpretation was made based solely on the group-specific correlation results.

## 4. Discussion

Growth factors play a key role in the success of RET, and dentin acts as a natural reservoir of bioactive molecules released during tooth development [14]. Because regenerative endodontic procedures depend not only on disinfection but also on the preservation and release of dentin-derived signaling molecules, evaluating how different irrigation protocols influence growth factor liberation is biologically relevant [19]. TGF-β1 is a critical regulator of odontoblastic differentiation and dentinogenesis through stimulation of dentin sialophosphoprotein and dentin matrix protein 1, while IGF-1 contributes to cell differentiation and mineralized tissue formation via increased alkaline phosphatase activity [25,33]. Accordingly, the present study focused on how different adjunctive ozone delivery protocols used in combination with chelation influenced the release of dentin-derived TGF-β1 and IGF-1 over time.

Previous studies investigating dentin-derived growth factor release have used different experimental models and normalization methods [16,34,35]. In the present study, in order to evaluate the release levels, conical root segments of standardized length were prepared, and all external root surfaces were coated with nail varnish, leaving only the internal root dentin exposed. Moreover, the internal dentin surface was sealed in the negative control group. The absence of detectable growth factor values in the negative control group supports the interpretation that the measurable values in the experimental groups were mainly associated with the exposed intracanal dentin surface. In addition, internal dentin surface area was quantified by micro-CT, and growth factor release was expressed as ng/mm^2^. In most previous studies, growth factor concentrations were reported using volume-based units or root canal volume-based normalization approaches (ng/mL) [30,32]. In contrast, the present study quantified growth factor release per unit dentin surface area (ng/mm^2^) using micro-CT. Because the measured growth factors are released from the exposed dentin surface rather than from the canal volume itself, surface-area normalization was used to account for interspecimen differences in the available dentin surface area. This approach was intended to provide a more standardized experimental assessment of growth factor release; however, it should not be interpreted as direct evidence of greater clinical relevance.

Widbiller et al. [31] reported that ultrasonic activation during irrigation increased TGF-β1 release from dentin discs. Similarly, Hancerliogulları et al. [30] showed that EDTA combined with ultrasonic activation significantly increased TGF-β1 levels on day 7. In the present study, the directly observed finding was that TGF-β1 increased significantly over time in the standard EDTA group and in the ozonated EDTA followed by ozone gas group, whereas no significant temporal increase was observed in the EDTA followed by ozonated water and ozone gas group. However, unlike Hancerliogulları et al. [30], no significant increase in IGF-1 levels was observed. This difference may reflect factor-specific release behavior rather than a uniform response of all dentin-derived bioactive molecules to chelation and adjunctive ozone application. TGF-β1 is stored in the dentin matrix in a latent form and may be more readily affected by demineralization, matrix exposure, or oxidative conditions. In contrast, IGF-1 is largely associated with IGF-binding proteins and may show different matrix-binding characteristics, release kinetics, and detectability under the same experimental conditions. The relatively low IGF-1 values observed in the present study may also have limited the ability to detect small protocol-related changes. Therefore, the absence of a significant IGF-1 increase should be interpreted cautiously and may indicate that the tested protocols had a more pronounced effect on measurable TGF-β1 than on IGF-1. It should also be noted that TGF-β1 increased significantly over time not only in the ozonated EDTA followed by ozone gas group but also in the standard irrigation group. This finding suggests that chelation under the present experimental conditions was itself sufficient to promote measurable TGF-β1 release, whereas adjunctive ozone appeared to modify this response in a protocol-dependent manner rather than generate an entirely distinct effect. At the same time, because all specimens underwent activation, the present findings do not allow separate conclusions regarding the isolated contribution of activation itself.

While ozone has been extensively investigated for its antimicrobial effects and clinical applications, its combination with EDTA and its influence on dentin-derived growth factor release have not been previously explored [26,36,37]. Therefore, the present study should be interpreted as an experimental evaluation of whether adjunctive ozone delivery protocols can modify measurable dentin-derived growth factor release when used in combination with chelation, rather than as evidence of a confirmed biostimulatory mechanism. Ozone was selected not because its antimicrobial effect alone implies enhanced growth factor release, but because its oxidative reactivity and reported biological effects suggested that it could potentially influence the measurable release or detectability of dentin-associated signaling molecules when used adjunctively with chelation. However, this proposed mechanism remains hypothetical and should be distinguished from the observed biochemical findings.

Evidence from medical and oral tissue engineering literature indicates that ozone can modulate growth factors involved in tissue repair and angiogenesis [26,38,39]. In the present study, TGF-β1 release increased significantly over time in the group treated with ozonated EDTA followed by ozone gas, and the day 7 TGF-β1 level in this group was significantly higher than that in the group treated with EDTA followed by ozonated water and ozone gas. Although EDTA facilitates demineralization and exposure of matrix-bound proteins, combining it with an oxidizing agent may alter the local chemical environment in ways that could affect the stability, availability, or detectability of released molecules. Together, these findings suggest that the effect of ozone on measurable dentin-derived TGF-β1 release may depend on the sequence and form of ozone application. The lower TGF-β1 values observed in the EDTA followed by ozonated water and ozone gas group may indicate possible protocol-dependent chemical interactions between sequential application steps. However, EDTA stability, chelating capacity, pH, and ozone-related oxidative changes were not directly evaluated; therefore, chemical antagonism cannot be confirmed or excluded. These findings should be interpreted as protocol-dependent biochemical outcomes, and future studies should chemically characterize EDTA-ozone interactions.

In addition to possible chemical interactions, the potential influence of combined EDTA-ozone protocols on the mechanical properties of dentin should also be considered. EDTA-mediated demineralization may reduce dentin microhardness and alter the mineralized surface, while ozone-derived oxidative species may theoretically interact with the organic matrix, including collagen. Therefore, the combined use of chelation and ozone may have consequences for dentin surface integrity, microhardness, and root resistance, particularly in immature teeth with thin dentinal walls. Because the present study did not evaluate dentin microhardness, surface erosion, collagen integrity, or fracture resistance, the findings cannot be used to confirm the mechanical safety of the tested protocols. Future studies should combine growth factor analysis with mechanical and ultrastructural assessments of dentin to determine whether the biochemical effects of combined EDTA-ozone protocols are accompanied by unfavorable changes in dentin structure or strength.

The observed increase in measurable TGF-β1 may be partly related to interactions between ozone-derived reactive oxygen species, EDTA-conditioned dentin, and matrix-associated proteins. However, direct evidence demonstrating ozone-mediated release or activation of matrix-bound TGF-β1 from human dentin remains limited; therefore, this mechanism should be considered theoretical rather than established. The clinical magnitude of this statistically significant difference should also be interpreted cautiously. Although the ozonated EDTA followed by ozone gas protocol produced higher surface-area-normalized TGF-β1 values on day 7, the absolute amount of released TGF-β1 remained low and was measured under controlled in vitro conditions. Therefore, this difference should be regarded as a protocol-dependent biochemical finding rather than direct evidence of clinically meaningful regenerative efficacy. Moreover, an increase in TGF-β1 does not necessarily indicate a beneficial regenerative response. The biological effects of TGF-β1 are context-, concentration-, and cell-dependent, and excessive or dysregulated TGF-β1 signaling may also be associated with fibrotic responses or undesirable mineralized tissue formation. Therefore, the present findings should be interpreted as evidence of altered measurable TGF-β1 release, rather than as proof of a favorable regenerative outcome. Whether this magnitude of TGF-β1 increase is sufficient to influence stem cell behavior, dentin–pulp tissue repair, or clinical RET outcomes requires confirmation through complementary cell-based, animal, and clinical studies.

The biological effects of ozone primarily occur through reactive oxygen species (ROS). It has been demonstrated in various systems that ROS can activate latent TGF-β1 complexes [40,41]. Krstić et al. [42] reported that ROS regulates TGF-β1 activation and signaling pathways; however, excessive ROS exposure may suppress receptor expression. In a recently published review, it was reported that ozone increases not only TGF-β1 but also growth factors such as VEGF and PDGF through ROS production, thereby supporting angiogenesis and tissue healing [43]. However, these findings were obtained in different biological systems and should not be directly extrapolated to dentin-derived growth factor release. In the present study, no mechanistic assay was performed to verify ozone-derived ROS activity, latent TGF-β1 activation within dentin, receptor-level signaling, or downstream regenerative responses. Therefore, the ROS-related explanation should be considered a theoretical biochemical hypothesis rather than a confirmed dentin-specific mechanism.

Accordingly, the increase observed in TGF-β1 levels in the ozonated EDTA followed by ozone gas group may be related to interactions between ozone-derived ROS and EDTA-conditioned dentin or matrix-associated proteins; however, the present study cannot determine whether this reflected increased release, activation, or altered detectability of latent TGF-β1. Thus, the present results should be interpreted as changes in measurable dentin-derived TGF-β1 values after the ELISA activation procedure, rather than as direct evidence that the ozone protocols activated latent TGF-β1 within the dentin matrix. Because smear layer removal, dentin ultrastructure, and surface roughness were not evaluated, the observed increase in measurable TGF-β1 values cannot be attributed exclusively to biological modulation. Physical surface changes induced by EDTA and/or ozone may also have influenced dentin matrix accessibility and the amount of growth factor detected in PBS. Therefore, these findings should be interpreted as protocol-dependent biochemical differences rather than evidence of a specific biological mechanism. In contrast, IGF-1 is mainly bound to IGF binding proteins and is not directly activated by ROS; moreover, ROS may suppress IGF-1 receptor signaling [44]. Thus, the absence of a significant change in IGF-1 levels may indicate that the tested ozone protocols did not uniformly affect all dentin-derived growth factors under the present experimental conditions.

The lack of increased TGF-β1 release in the EDTA followed by ozonated water and ozone gas group may reflect protocol-dependent differences in the timing and sequence of chelation and ozone exposure rather than a confirmed biological mechanism. One possible explanation is that sequential application may have reduced the effective interaction between reactive ozone species and dentin-bound bioactive components during the period of chelation-mediated matrix exposure. However, ozone stability, EDTA-ozone interactions, pH changes, and oxidative effects were not directly evaluated in the present study. Therefore, this interpretation should be regarded as a hypothesis rather than a demonstrated mechanism. Future studies should investigate whether different ozone delivery sequences alter EDTA stability, chelating activity, and dentin matrix accessibility.

The combined use of EDTA and ozone may also have potential drawbacks. Although EDTA facilitates demineralization and exposure of matrix-bound proteins, combining it with an oxidizing agent may alter the local chemical environment in ways that are not yet fully understood. Such interactions may affect the stability, availability, or biological activity of released molecules. In addition, the potential influence of combined EDTA-ozone protocols on the mechanical properties of dentin should be considered. EDTA-mediated demineralization may reduce dentin microhardness and alter the mineralized surface, while ozone-derived oxidative species may theoretically interact with the organic matrix, including collagen. Therefore, the combined use of chelation and ozone may have consequences for dentin surface integrity, microhardness, and root resistance, particularly in immature teeth with thin dentinal walls. Because the present study did not evaluate dentin microhardness, surface erosion, collagen integrity, or fracture resistance, the findings cannot be used to confirm the mechanical safety of the tested protocols. Therefore, the present findings should not be interpreted as evidence that the protocol using ozonated EDTA followed by ozone gas is universally advantageous, but rather as preliminary evidence that this adjunctive approach may differentially influence specific growth factors, particularly TGF-β1, under the tested in vitro conditions. Future studies should combine growth factor analysis with mechanical and ultrastructural assessments of dentin to determine whether the biochemical effects of combined EDTA-ozone protocols are accompanied by unfavorable changes in dentin structure or strength.

Despite the standardized experimental design, the in vitro nature of the present study limits direct clinical translation of the findings. The experimental model allowed controlled evaluation of dentin-derived growth factor release; however, it does not reproduce the biological complexity of clinical RET conditions, including immature periapical tissues, stem cell responses, inflammatory microenvironments, vascularization, or host-mediated healing. Additional limitations should also be acknowledged. First, ELISA provided an indirect quantitative assessment of released growth factors and did not determine whether the detected molecules remained biologically active. Second, although autoclave sterilization was not performed to avoid heat-related alteration of dentin structure and protein integrity, the absence of terminal sterilization means that the possible influence of residual biological remnants cannot be completely excluded. Third, the study evaluated only short-term release on day 1 and day 7 and therefore does not reflect the full temporal complexity of regenerative healing. A further limitation of the statistical approach is that formal treatment-by-time interaction effects were not directly estimated using a repeated-measures model. Instead, nonparametric within-group temporal comparisons and intergroup comparisons at each time point were performed because several variables did not meet the normality assumption and the sample size within each group was limited. Therefore, the temporal findings should be interpreted cautiously. Fourth, despite random allocation and standardized canal preparation, significant intergroup differences in root canal volume and internal dentin surface area were observed, likely reflecting residual anatomical variability among extracted human teeth. Although surface-area normalization was used to reduce the influence of exposed dentin surface differences, it may not fully compensate for baseline anatomical differences such as canal volume, dentin thickness, or three-dimensional canal geometry. Therefore, this imbalance should be considered when interpreting the results. Fifth, the biological safety of ozone application should be interpreted cautiously, particularly in RET involving immature periapical tissues. Although ozone-derived ROS may contribute to antimicrobial and biochemical effects, their biological impact may be dose- and exposure-dependent. Because the present study did not assess cytotoxicity, stem cell viability, inflammatory responses, or periapical tissue compatibility, the tested ozone protocols should not be directly extrapolated to clinical RET conditions without further biological safety validation. Finally, no cell-based, animal, or clinical validation was performed. Further cell-based, animal, and clinical studies using broader biomarker panels are needed to enhance translational relevance and to determine the biological safety and clinical applicability of ozone-assisted irrigation protocols in RET. Future studies may also evaluate adjunctive intracanal laser applications, alone or in combination with ozone-based protocols, for bacterial load reduction, dentin surface modification, and tissue repair-related outcomes [45]. However, because laser application was not evaluated in the present study, this should be considered only as a future research direction.

Overall, the present findings indicate that adjunctive ozone application did not produce a uniform increase in dentin-derived growth factor release. Rather, its influence appeared to be protocol-dependent and more evident for TGF-β1 than for IGF-1 under the present in vitro conditions. Within the tested protocols, ozonated EDTA followed by ozone gas was associated with higher measurable TGF-β1 values than EDTA followed by ozonated water and ozone gas, whereas IGF-1 levels remained unaffected by ozone application. However, these findings should be interpreted as preliminary biochemical observations and should not be considered evidence of superior regenerative efficacy or clinical advantage.

## 5. Conclusions

Adjunctive ozone application did not uniformly enhance dentin-derived growth factor release under the conditions of this in vitro study. Its effect was protocol-dependent and was observed mainly for TGF-β1, whereas IGF-1 release remained unaffected. Among the tested ozone protocols, ozonated EDTA followed by ozone gas was associated with higher measurable TGF-β1 values than EDTA followed by ozonated water and ozone gas. However, these findings represent in vitro biochemical changes and do not establish a clinically significant regenerative benefit. Further cell-based, animal, and clinical studies are required to determine the biological and clinical significance of these findings.

## Figures and Tables

**Table 1:** Distribution and comparison of surface-area-normalized IGF-1 and TGF-β1 release values across study groups and measurement time points.

IGF-1 Release Values							
	Day 1 Mean ± SD	Day 1 Median (Q1–Q3)	Day 7 Mean ± SD	Day 7 Median (Q1–Q3)	Within-Group Test Statistic	*p*-Value	Effect Size
Group A	0.06 ± 0.04	0.06 (0.04–0.07)	0.08 ± 0.03	0.07 (0.05–0.10)	−0.982	0.326	–
Group B	0.08 ± 0.07	0.06 (0.05–0.09)	0.07 ± 0.04	0.06 (0.04–0.08)	−0.414	0.679	–
Group C	0.09 ± 0.11	0.06 (0.04–0.09)	0.07 ± 0.02	0.07 (0.05–0.08)	−0.529	0.796	–
Total	0.08 ± 0.08	0.06 (0.04–0.09)	0.07 ± 0.03	0.06 (0.05–0.09)	−0.585	0.559	–
Intergroup test statistic	0.840		1.263				
Intergroup *p*-value	0.657		0.532				
**TGF-β1 Release Values**							
	**Day 1 Mean ± SD**	**Day 1 Median (Q1–Q3)**	**Day 7 Mean ± SD**	**Day 7 Median (Q1–Q3)**	**Within-Group Test Statistic**	***p*-Value**	**Effect Size**
Group A	0.02 ± 0.02	0 (0–0.05)	0.06 ± 0.04	0.05 (0.03–0.10)	−2.275	0.023 *	0.680
Group B	0.04 ± 0.06	0 (0–0.07)	0.03 ± 0.03	0.02 (0.01–0.06)	−0.207	0.836	–
Group C	0.03 ± 0.04	0.01 (0–0.05)	0.09 ± 0.07	0.07 (0.05–0.12)	−2.947	0.003 *	0.879
Total	0.03 ± 0.05	0 (0–0.05)	0.06 ± 0.05	0.05 (0.02–0.08)	−3.244	0.001 *	0.472
Intergroup test statistic	0.305		7.608				
Intergroup *p*-value	0.859		0.022 *				
Intergroup effect size	–		0.171				

* *p* < 0.05; SD: standard deviation; Q1: first quartile; Q3: third quartile.

**Table 2:** Distribution and comparison of micro-CT-derived root canal volume and internal dentin surface area across study groups.

	Volume (mm^3^)		Surface Area (mm^2^)	
	Mean ± SD	Median (Q1–Q3)	Mean ± SD	Median (Q1–Q3)
Group A	9.36 ± 1.15	9.38 (8.51–10.32)	41.97 ± 5.55	42.09 (37.72–45.75)
Group B	11.21 ± 1.13	10.99 (10.53–12.19)	52.61 ± 9.35	52.58 (46.9–54.69)
Group C	10.56 ± 1.77	10.13 (9.85–10.87)	50.91 ± 14.71	46.82 (43.25–53.57)
Intergroup test statistic	14.702		14.071	
Intergroup *p*-value	<0.001 *		<0.001 *	

* *p* < 0.05; SD: standard deviation; Q1: first quartile; Q3: third quartile.

**Table 3:** Exploratory pooled Spearman correlations between IGF-1 and TGF-β1 values at different time points.

		IGF-1 Day 7	TGF-β1 Day 1	TGF-β1 Day 7
IGF-1 Day 1	r	0.040	0.478	−0.035
	*p*	0.787	0.001 *	0.814
IGF-1 Day 7	r		0.250	0.278
	*p*		0.087	0.056
TGF-β1 Day 1	r			0.413
	*p*			0.004 *

* *p* < 0.05 and r: correlation coefficient.

**Table 4:** Exploratory group-specific Spearman correlations between IGF-1 and TGF-β1 values at different time points.

Group A		IGF-1 Day 7	TGF-β1 Day 1	TGF-β1 Day 7
IGF-1 Day 1	r	−0.103	0.675	−0.318
	*p*	0.704	0.004 *	0.231
IGF-1 Day 7	r		0.252	0.221
	*p*		0.347	0.412
TGF-β1 Day 1	r			−0.052
	*p*			0.850
**Group B**		**IGF-1 Day 7**	**TGF-β1 Day 1**	**TGF-β1 Day 7**
IGF-1 Day 1	r	0.135	0.209	0.024
	*p*	0.617	0.438	0.931
IGF-1 Day 7	r		0.138	−0.041
	*p*		0.610	0.880
TGF-β1 Day 1	r			0.803
	*p*			<0.001 *
**Group C**		**IGF-1 Day 7**	**TGF-β1 Day 1**	**TGF-β1 Day 7**
IGF-1 Day 1	r	0.153	0.621	0.215
	*p*	0.572	0.010 *	0.425
IGF-1 Day 7	r		0.306	0.665
	*p*		0.249	0.005 *
TGF-β1 Day 1	r			0.579
	*p*			0.019 *

* *p* < 0.05 and r: correlation coefficient.

## Data Availability

The original data presented in this study are included in the article. Additional inquiries may be directed to the corresponding author.

## Abbreviations

The following abbreviations are used in this manuscript:

RET	Regenerative endodontic treatment
EDTA	Ethylenediaminetetraacetic acid
TGF-β1	Transforming growth factor beta 1
IGF-1	Insulin-like growth factor 1
NaOCl	Sodium hypochlorite
PRILE	Preferred Reporting Items for Laboratory studies in Endodontology
micro-CT	Micro-computed tomography
PBS	Phosphate-buffered saline
ELISA	Enzyme-Linked Immunosorbent Assay
Q1	First quartile
Q3	Third quartile
SD	Standard deviation
ROS	Reactive oxygen species
